# The Selective NMDA Receptor GluN2B Subunit Antagonist CP-101,606 with Antidepressant Properties Modulates Cytochrome P450 Expression in the Liver

**DOI:** 10.3390/pharmaceutics13101643

**Published:** 2021-10-09

**Authors:** Ewa Bromek, Anna Haduch, Marta Rysz, Joanna Jastrzębska, Renata Pukło, Olga Wójcikowska, Przemysław Jan Danek, Władysława Anna Daniel

**Affiliations:** Department of Pharmacokinetics and Drug Metabolism, Maj Institute of Pharmacology, Polish Academy of Sciences, 31-343 Kraków, Poland; bromek@if-pan.krakow.pl (E.B.); haduch@if-pan.krakow.pl (A.H.); marta.rysz@gmail.com (M.R.); czyzyk@if-pan.krakow.pl (J.J.); bartyzel@if-pan.krakow.pl (R.P.); olgawojcikowska98@gmail.com (O.W.); danek@if-pan.krakow.pl (P.J.D.)

**Keywords:** CP-101,606, NMDA receptor, cytochrome P450, liver, neuroendocrine regulation

## Abstract

Recent research indicates that selective NMDA receptor GluN2B subunit antagonists may become useful for the treatment of major depressive disorders. We aimed to examine in parallel the effect of the selective NMDA receptor GluN2B subunit antagonist CP-101,606 on the pituitary/serum hormone levels and on the regulation of cytochrome P450 in rat liver. CP-101,606 (20 mg/kg ip. for 5 days) decreased the activity of CYP1A, CYP2A, CYP2B, CYP2C11 and CYP3A, but not that of CYP2C6. The alterations in enzymatic activity were accompanied by changes in the CYP protein and mRNA levels. In parallel, a decrease in the pituitary growth hormone-releasing hormone, and in serum growth hormone and corticosterone (but not T_3_ and T_4_) concentration was observed. After a 3-week administration period of CP-101,606 less changes were found. A decrease in the CYP3A enzyme activity and protein level was still maintained, though no change in the mRNA level was found. A slight decrease in the serum concentration of corticosterone was also maintained, while GH level returned to the control value. The obtained results imply engagement of the glutamatergic system in the neuroendocrine regulation of cytochrome P450 and potential involvement of drugs acting on NMDA receptors in metabolic drug–drug interactions.

## 1. Introduction

Knowledge of the regulation of liver cytochrome P450 by drugs and toxic substances at the level of the hepatocyte is already broad, however, physiological regulation of its expression, especially the role of the nervous system has been the subject of research only for the last decade. Since secretion of hormones regulating cytochrome P450 genes (e.g., growth hormone, corticosterone and thyroid hormones) is under the control of the brain nervous system (mostly by the hypothalamus), therefore, the changes in brain neurotransmission can affect endocrine regulation of cytochrome P450 in the liver. The neuroendocrine regulation of cytochrome P450 has already been shown to occur via the brain dopaminergic [1,2,3], noradrenergic [4,5,6] and serotonergic [7,8,9,10,11,12,13] systems.

A potential involvement of the glutamatergic system in the regulation of cytochrome P450 expression has not been examined so far. Immune–histochemical studies have shown that the hypothalamic paraventricular and arcuate nuclei are deeply innervated by the glutamatergic system and are rich in all subtypes of glutamate receptors [14,15,16,17]. There is a dense innervation of growth hormone-releasing hormone (GHRH)-containing neurons in the arcuate nuclei, as well as of corticotropin-releasing hormone (CRH), thyrotropin-releasing hormone (TRH) and somatostatin synthesizing-neurons in the hypothalamic paraventricular nuclei. Furthermore, glutamatergic innervation of the hypothalamic median eminence arising from different hypothalamic structures has been described [18,19]. Ionotropic (NMDA, AMPA and KA) and metabotropic (mGlu) receptors, are observed pre- and post-synaptically in the above-mentioned hypothalamic structures and the pituitary [20,21,22,23]. Functional glutamate receptors have also been found on anterior pituitary hormone-producing cells [14,22,24]. Moreover, glutamate receptors have been shown to be engaged in the regulation of secretion of hypothalamic/pituitary hormones [25,26,27,28]. The abovementioned anatomic (receptor mapping) and physiological characteristics of glutamate receptors imply that they may take part in the central neuroendocrine regulation of hepatic cytochrome P450.

Ketamine, a non-subunit-selective NMDA receptor antagonist, shows a rapid and potent antidepressant activity, but it produces serious side-effects in the central nervous system [29] and affects cytochrome P450 in the liver [30]. When administered at high doses, ketamine induces cytochrome P450 enzymes.

Therefore, many other NMDA receptor-based agents have been developed, such as NMDA receptor subunit antagonists, to provide safer antidepressants. Recent research indicates that selective NMDA receptor GluN2B subunit antagonists, NMDA receptor glycine site partial agonists and mGlu_5_ negative allosteric modulators may become useful for the treatment of major depressive disorders [31,32,33,34,35]. However, it is of great concern that apart from their targeted therapeutic effect, new drugs may seriously affect neuroendocrine regulation of different physiological processes including the expression and activity of cytochrome P450.

The aim of this work was to examine whether the selective antagonist of GluN2B subunit of NMDA receptor, the compound CP-101,606 [36] showing antidepressant properties [37,38,39] affects liver cytochrome P450. A possible involvement of the neuroendocrine system in the regulation of cytochrome P450 by CP-101,606 has also been investigated, since NMDA receptor and its subunits have not been tested in this aspect before. The experiment was performed after subchronic (5 days) and chronic (3 weeks) treatment of male rats with pharmacological doses of CP-101,606. The expression and activity of cytochrome P450 enzymes, which are regulated hormonally and are involved in drug metabolism were investigated in the liver. Simultaneously, hormone levels were determined in the pituitary and blood serum.

## 2. Results

The effect of 5-day and 3-week treatment with the selective NMDA receptor GluN2B subunit antagonist CP-101,606 on cytochrome P450 enzymes activity was studied in rat liver. When the enzyme activity was changed, the effect of CP-101,606 on the enzyme expression was tested to find the mechanism of regulation.

### 2.1. The Effect of CP-101,606 on the CYP1A Activity and Expression in Rat Liver after 5-Day and 3-Week Treatment

**Figure 1:**
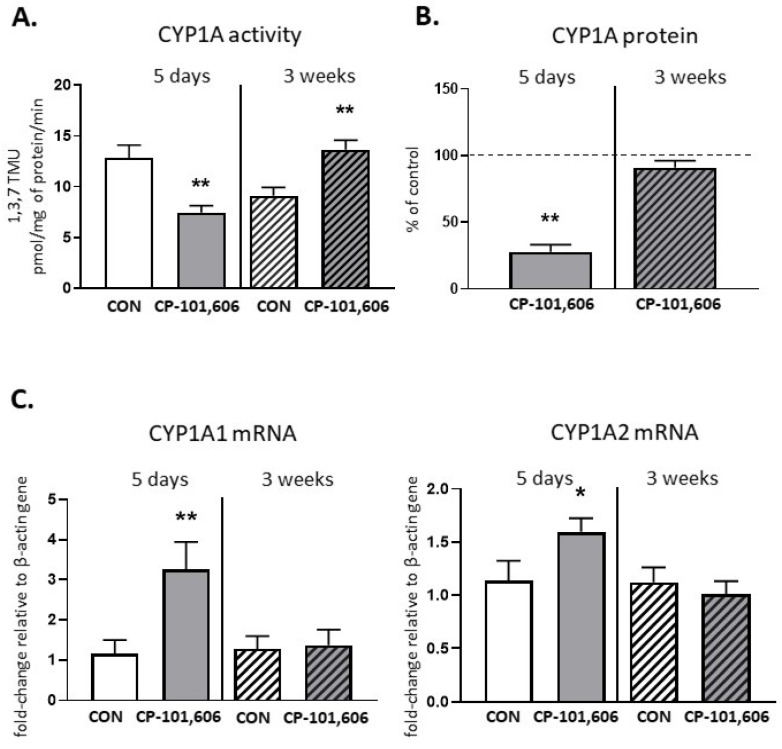
The effect of a 5-day or 3-week administration of a selective NMDA receptor GluN2B subunit antagonist (CP-101,606) on the cytochrome P450 1A (CYP1A) enzyme activity assessed as the rates of caffeine 8-hydroxylation (**A**), protein level (**B**) and mRNA (**C**). All values are shown as the mean ± S.E.M. Student’s *t*-test: * *p* < 0.05; ** *p* < 0.01, compared to the control (CON). The representative results of the Western immunoblot analysis (CYP1A protein bands) are shown in Figure 7.

The activity of CYP1A, measured as the rate of caffeine C-8-hydroxylation, decreased to 68.6% of the control value after 5-day treatment with CP-101,606 (Figure 1). The enzyme protein level was also significantly lowered compared to the control (down to 30% of the control value). However, the *CYP1A1* and *CYP1A2* mRNA levels significantly increased. In contrast to 5-day treatment, a 3-week treatment with CP-101,606 enhanced the CYP1A activity (up to 156% of the control value), though no change in the CYP1A protein level or *CYP1A1* and *CYP1A2* mRNA levels were observed (Figure 1).

### 2.2. The Effect of CP-101,606 on the CYP2A Activity and Expression in Rat Liver after 5-Day and 3-Week Treatment

**Figure 2:**
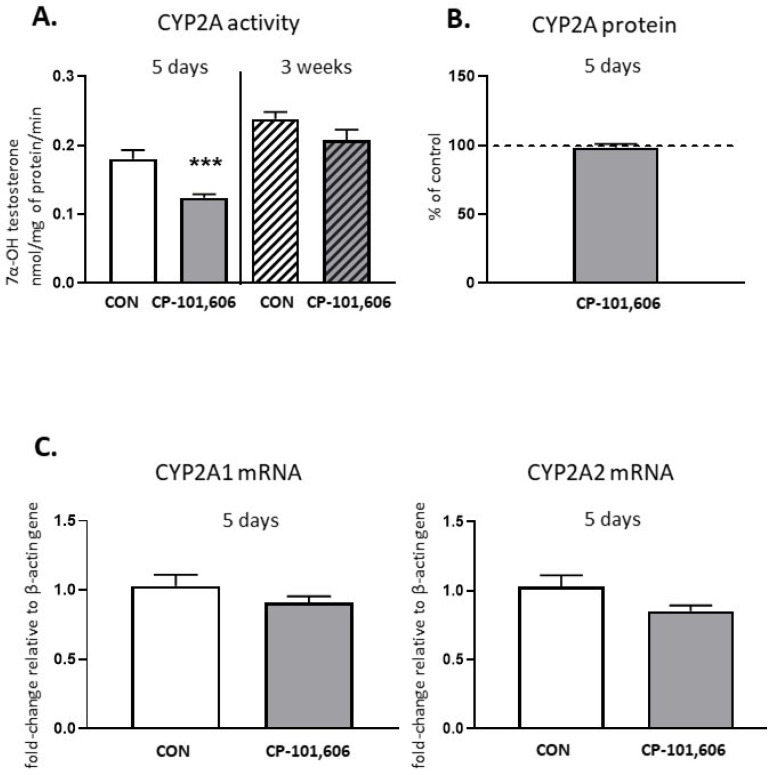
The effect of a 5-day or 3-week administration of a selective NMDA receptor GluN2B subunit antagonist (CP-101,606) on the cytochrome P450 2A (CYP2A) enzyme activity assessed as the rate of testosterone 7-α-hydroxylation (**A**), protein level (**B**) and mRNA (**C**). All values are shown as the mean ± S.E.M. Student’s *t*-test: *** *p* < 0.001, compared to the control (CON). The representative results of the Western immunoblot analysis (CYP2A protein bands) are shown in Figure 7.

The activity of CYP2A, estimated as the rate of the 7-α-hydroxylation of testosterone, declined to 68.7% of the control after 5-day treatment with CP-101,606, but no changes in the CYP2A protein level or *CYP2A1* and *CYP2A2* mRNA levels were observed (Figure 2). The CYP2A activity reduced by 5-day treatment returned to the control value after 3 weeks of treatment with CP-101,606.

### 2.3. The Effect of CP-101,606 on the CYP2B Activity and Expression in Rat Liver after 5-Day and 3-Week Treatment

**Figure 3:**
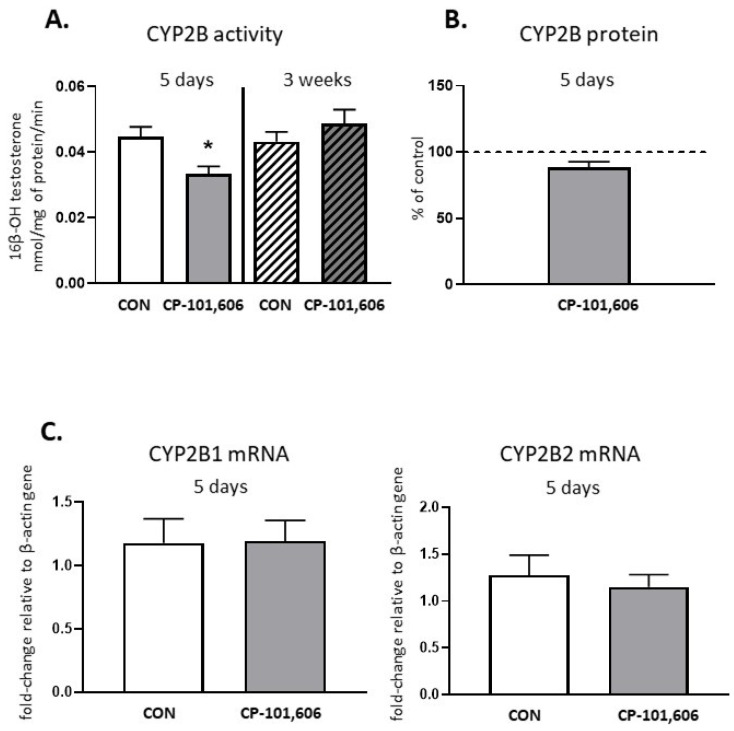
The effect of a 5-day or 3-week administration of a selective NMDA receptor GluN2B subunit antagonist (CP-101,606) on the cytochrome P450 2B (CYP2B) enzyme activity assessed as the rate of testosterone 16-β-hydroxylation (**A**), protein level (**B**) and mRNA (**C**). All values are shown as the mean ± S.E.M. Student’s *t*-test: * *p* < 0.05, compared to the control (CON). The representative results of the Western immunoblot analysis (CYP2B protein bands) are shown in Figure 7.

The activity of CYP2B, evaluated as the rate of the 16β-hydroxylation of testosterone, was diminished to 74.8% of the control after 5-day treatment with CP-101,606, though no change in the CYP2B protein or *CYP2B1* and *CYP2B2* mRNA levels were observed (Figure 3). The CYP2B activity diminished by 5-day treatment returned to the control value after a 3 week-treatment with CP-101,606.

### 2.4. The Effect of CP-101,606 on the CYP2C6 Activity and Expression in Rat Liver after 5-Day and 3-Week Treatment

**Figure 4:**
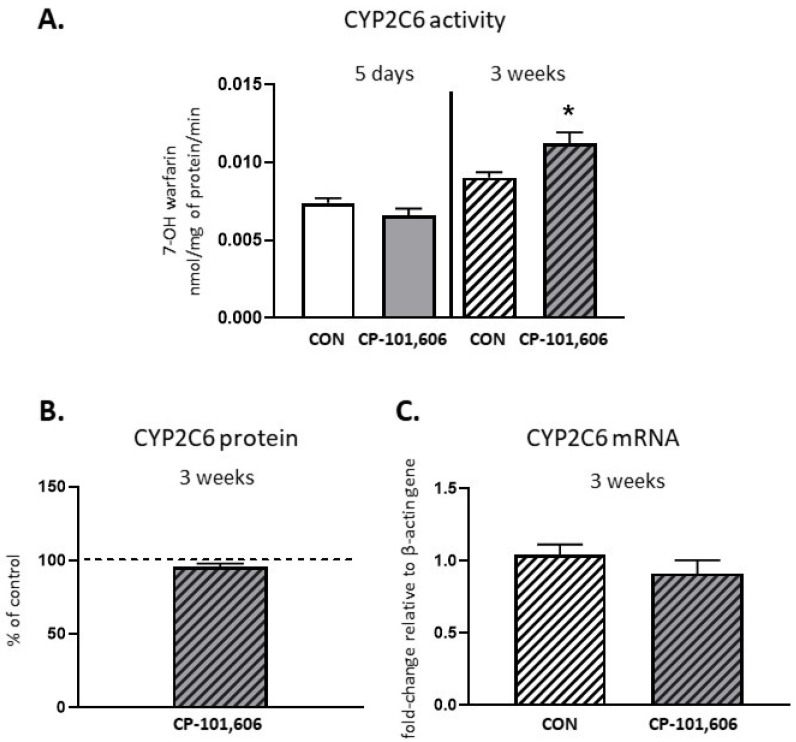
The effect of a 5-day or 3-week administration of a selective NMDA receptor GluN2B subunit antagonist (CP-101,606) on the cytochrome P450 2C6 (CYP2C6) enzyme activity assessed as the rate of warfarin 7-hydroxylation (**A**), protein level (**B**) and mRNA (**C**). All values are shown as the mean ± S.E.M. Student’s *t*-test: * *p* < 0.05, compared to the control (CON). The representative results of the Western immunoblot analysis (CYP2C6 protein bands) are shown in Figure 7.

The activity of CYP2C6, measured as a rate of the 7-hydroxylation of warfarin, was not significantly changed by 5-day treatment, and only increased (up to 118% of the control value) after 3 weeks of treatment with CP-101,606 (Figure 4). The CYP2C6 protein or *CYP2C6* mRNA level were not changed after short- or long-term treatment with the investigated compound.

### 2.5. The Effect of CP-101,606 on the CYP2C11 Activity and Expression in Rat Liver after 5-Day and 3-Week Treatment

**Figure 5:**
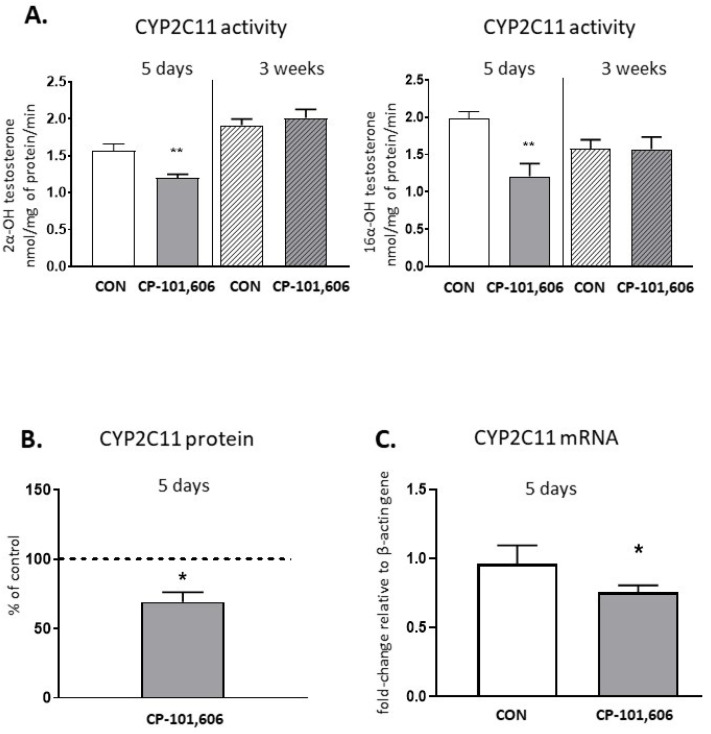
The effect of a 5-day or 3-week administration of a selective NMDA receptor GluN2B subunit antagonist (CP-101,606) on the cytochrome P450 2C11 (CYP2C11) enzyme activity assessed as the rate of testosterone 2-α- and 16-α-hydroxylation (**A**), protein level (**B**) and mRNA (**C**). All values are shown as the mean ± S.E.M. Student’s *t*-test: * *p* < 0.05; ** *p* < 0.01, compared to the control (CON). The representative results of the Western immunoblot analysis (CYP2C11 protein bands) are shown in Figure 7.

The activity of CYP2C11, tested as a rate of the 2-α- or 16-α-hydroxylation of testosterone, was reduced to 76.7% and 60.9% of the control value, respectively, after 5-day treatment with CP-101,606 (Figure 5). The CYP2C11 protein level was also significantly lowered compared to the control (down to 69% of the control value). Accordingly, the *CYP2C11* mRNA level significantly decreased after 5-day treatment with CP-101,606. The reduced activity of CYP2C11 by 5-day treatment returned to the control value after 3 weeks of treatment with CP-101,606.

### 2.6. The Effect of CP-101,606 on the CYP3A Activity and Expression in Rat Liver after 5-Day and 3-Week Treatment

**Figure 6:**
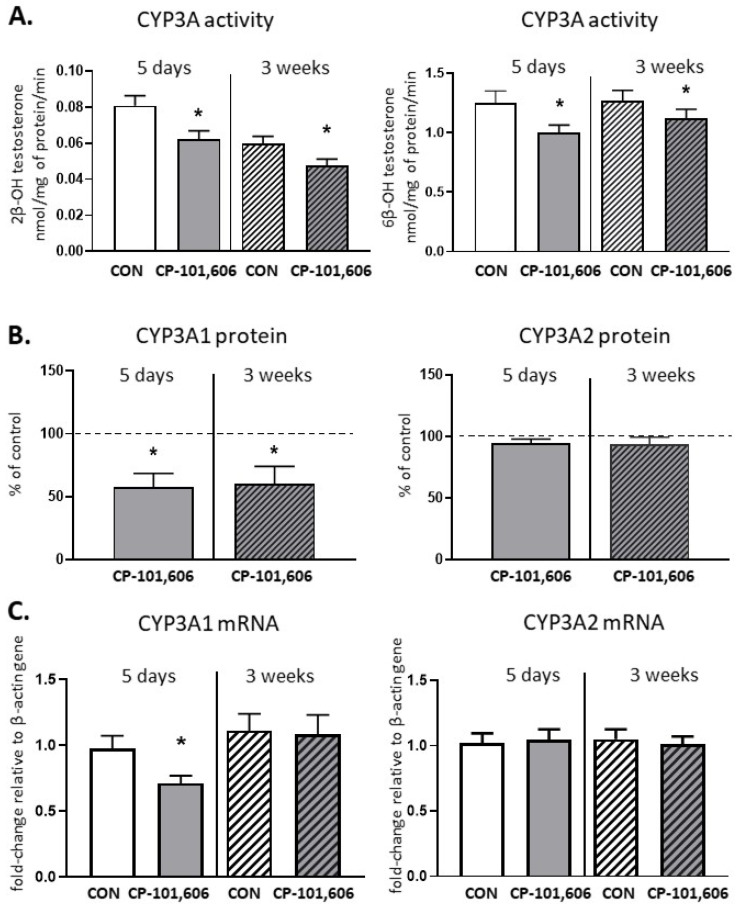
The effect of a 5-day or 3-week administration of a selective NMDA receptor GluN2B subunit antagonist (CP-101,606) on the cytochrome P450 3A (CYP3A) enzyme activity assessed as the rate of testosterone 2-β- and 6-β-hydroxylation (**A**), protein level (**B**) and mRNA (**C**). All values are shown as the mean ± S.E.M. Student’s *t*-test: * *p* < 0.05, compared to the control (CON). The representative results of the Western immunoblot analysis (CYP3A1 and CYP3A2 protein bands) are shown in Figure 7.

The CYP3A activity, determined as the rate of the 2-β- or 6-β-hydroxylation of testosterone, decreased to 77% and 79.8% of the control value, respectively, after 5-day treatment with CP-101,606 (Figure 6). The protein level of CYP3A1 was also significantly reduced compared to the control (down to 58% of the control value) and the *CYP3A1* mRNA level decreased as well after 5-day treatment. The decreased CYP3A activity (down to 61% and 73% of the control) and CYP3A1 protein level (down to 60% of the control) were still maintained after 3 weeks of treatment with CP-101,606, though the *CYP3A1/2* mRNA level remained unchanged.

**Figure 7:**
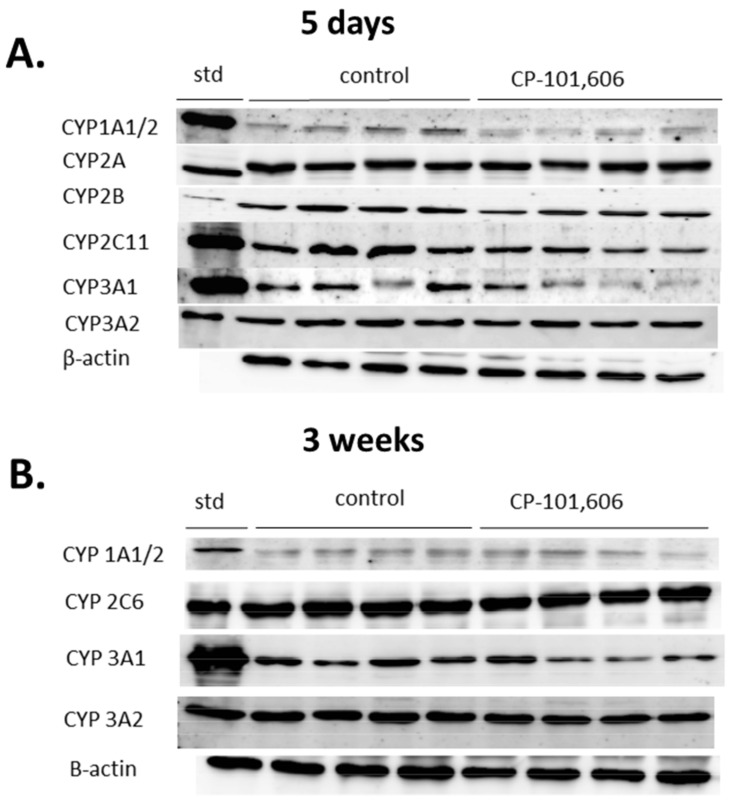
The effect of a 5-day (**A**) or 3-week (**B**) treatment with CP-101,606 on the intensity of cytochrome P450 protein bands (CYP1A, 2A, 2B, C6, 2C11, 3A1 and 3A2) in liver microsomes. Rat cDNA-expressed CYP enzymes were used as standards. The representative CYP protein bands of the Western immunoblot analysis are shown. The presented results are typical of four separate male rats per group. The mean values ± S.E.M. of CYP protein levels are shown in Figure 1B, Figure 2B, Figure 3B, Figure 4B, Figure 5B and Figure 6B. STD, standard.

The representative CYP protein bands in CP-101,606-treated rats, obtained as a result of the Western immunoblot analysis, are shown in Figure 7A (5-day treatment) and Figure 7B (3-week treatment).

### 2.7. The Effect of CP-101,606 on Pituitary and Serum Hormone Levels

**Figure 8:**
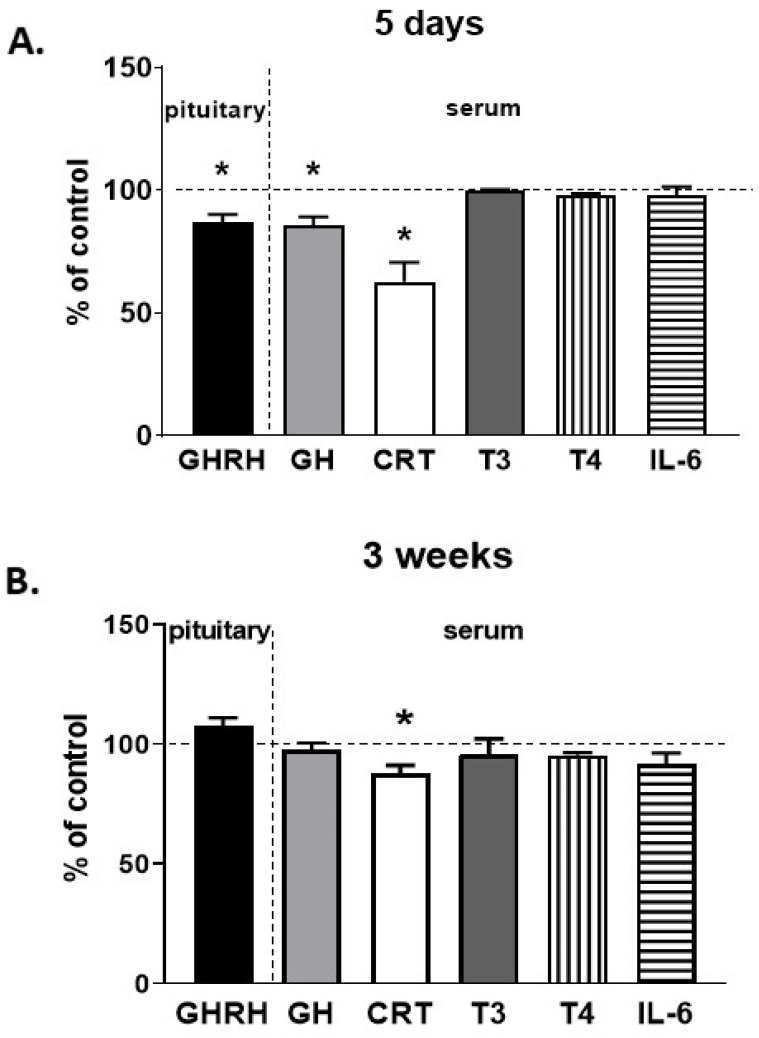
The influence of a selective NMDA receptor GluN2B subunit antagonist (CP-101,606) on the pituitary GHRH level and serum hormone concentrations after 5-day (A) and 3-week (B) treatment. All values are presented as the mean ± S.E.M. The absolute control values for the 5-day experiment were: 49.45 ± 3.3 pg/mg for GHRH in the pituitary and 1.25 + 0.05, 112.5 ± 17.35, 6.7 ± 0.04, 62 ± 0.4 and 12.6 ± 0.38 ng/mL for serum GH, CRT, T3, T4, IL-6 (respectively). The absolute control values for 3-week experiment were: 93.11 ± 5.9 pg/mg for GHRH in the pituitary and 4.47 + 0.09, 8.06 ± 0.3, 4.2 ± 0.013, 22.9 + 0.3 ng/mL and 3.2 + 0.08 ng/mL for serum GH, CRT, T3, T4, IL-6 (respectively). Student’s *t*-test: * *p* < 0.05, compared to the control (CON). GHRH, growth hormone-releasing hormone; GH, growth hormone; CRT, corticosterone.

After 5-day treatment with CP-101,606, the level of growth hormone-releasing hormone (GHRH) in the pituitary was significantly lower compared to control animals (Figure 8A). At the same time, the serum concentration of growth hormone (GH) and corticosterone (CRT) declined, while the levels of the thyroid hormones (T_3_ and T_4_) and interleukin 6 (IL-6) were unaffected.

After 3-week treatment, a gentle diminution in the serum concentration of corticosterone was still maintained, while that of GH returned to the control value (Figure 8B).

## 3. Discussion

A possible role of the neuroendocrine regulation of cytochrome P450 expression involving the glutamatergic system has not yet been studied. Our study indicates that apart from the brain monoaminergic systems, the brain glutamatergic system may also affect the cytochrome P450 expression and activity via NMDA receptors and endocrine mechanisms.

CP-101,606, a selective NMDA receptor GluN2B subunit antagonist, produced a lot of changes in the activities of cytochrome P450 enzymes in rat liver after 5-day treatment, as summarized in Table 1.

A decrease in the activity of CYP1A, 2A, 2B, 2C11 and 3A, but not in CYP2C6 was observed. The investigated CYP isoforms are regulated by hormones, such as growth hormone (GH), glucocorticoids (corticosterone) and thyroid hormones (triiodothyronine T_3_ and thyroxine T_4_) and via activation of the respective membrane, cytoplasmic or nuclear receptors, in this way regulating the transcription of *CYP* genes [40,41,42,43,44,45,46].

The detailed analysis of the influence of glutamate receptor agonists and antagonists on GH release has shown that glutamate can elicit prominent and dual regulatory responses on GH secretion, involving a predominantly stimulatory effect via ionotropic receptors, as well as a minor inhibitory effect via metabotropic receptors [28]. Thus, the activation of NMDA receptors resulted in the stimulation of GH secretion; however, the stimulus did not originate in the pituitary, but was generated at the level of GHRH and/or somatostatin neurons. In accordance with the abovementioned neuroendocrine pathway, a decrease in the GHRH level and GH-related expression (mRNA and protein) and activity of CYP2C11 in male rat liver was observed after a 5-day treatment with the selective NMDA receptor GluN2B subunit antagonist CP-101,606 in our experiment. GH is the main regulator of CYP2C11, as well as one of the regulators of other CYP enzymes [40], and our previous studies indicated a positive correlation between the activities of CYP2C11 and CYP3A on the one hand, and the serum GH concentration on the other in male rats [6]. Alternatively, an interplay of the glutamatergic system with other neuroendocrine regulators of GH release, such as the monoamine neurotransmitters dopamine, noradrenaline or serotonin seems also possible. NMDA receptors were shown to be present on catecholaminergic and indoleaminergic neurons and to affect the function of the monoaminergic systems in the brain [47,48]. An important role of the mentioned monoaminergic systems in the GH regulation has been documented [4,5,13,49]. On the other hand, the expression of CYP2C6 enzyme, which is less susceptible to neuroendocrine regulation, was not affected by a 5-day treatment with the investigated compound.

**Figure 9:**
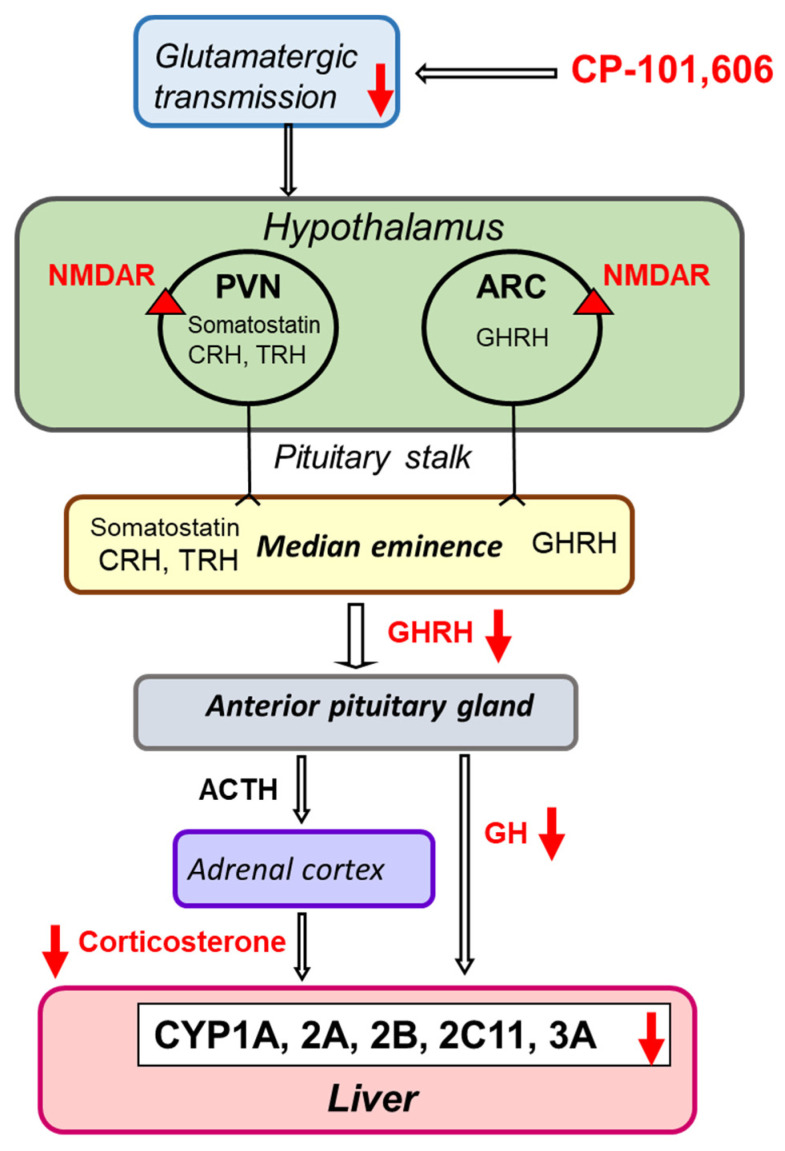
A hypothetical mechanism of neuroendocrine regulation of liver cytochrome P450 by the selective NMDA receptor GluN2B subunit antagonist CP-101,606. Through its antagonistic effect on NMDA receptors (NMDARs) in the paraventricular (PVN) and arcuate (ARC) nuclei of the hypothalamus, CP-101,606 negatively regulates growth hormone (GH) and corticosterone secretion and, in turn, the cytochrome P450 expression in the liver. CRH, corticotropin-releasing hormone; TRH, thyrotropin-releasing hormone; GHRH, growth hormone-releasing hormone; ACTH, adrenocorticotropic hormone.

There is also strong evidence that glutamate drives the hypothalamo–pituitary–adrenal (HPA) axis stress responses through postsynaptic ionotropic glutamate receptors (NMDA and non-NMDA) localized on neurons in the hypothalamic paraventricular nuclei [27]. Accordingly, a decrease in the serum concentration of corticosterone and corticosterone-related expression and activity of liver CYP3A was observed after a 5-day treatment with the selective NMDA receptor GluN2B subunit antagonist in our experiment. The hypothetical mechanism of the observed changes in CYP enzyme activities after subchronic treatment with CP-101,606 is presented in Figure 9.

In the case of other investigated cytochrome P450 enzymes, a correlation between the activity, protein and mRNA level was less pronounced, which may suggest involvement of other mechanisms, such as posttranscriptional regulation of CYP1A (an increase in *CYP1A1/2* mRNA, but a decrease in the CYP1A protein and activity) or posttranslational modification of CYP2A and CYP2B protein (an increase in enzyme activity at no change in the enzyme protein and mRNA) after a 5-day treatment with CP-101,606.

After a 3-week administration of the selective NMDA receptor GluN2B subunit antagonist CP-101,606 less changes in the investigated enzymes were found, probably as a result of adaptive mechanisms. Thus, the decreased serum concentration of GH, observed after a 5-day treatment, returned to the control value, while that of corticosterone was still slightly diminished. A decrease in the CYP3A1 enzyme activity and protein level, observed after a 5-day treatment, was maintained at a similar level after a 3-week treatment; however, no change in the mRNA level was observed, which suggest a posttranscriptional mechanism of the enzyme regulation after prolonged treatment with CP-101,606. Interestingly, the CYP1A activity increased (at no change in the enzyme protein and mRNA), which is contrary to the result observed after 5-day treatment. This may also be caused by some adaptive changes in the functioning of CYP1A protein that developed during prolonged administration of CP-101,606. Neuroimmune mechanisms do not seem to be involved in the effects produced by CP-101,606 on CYP enzymes, since the level of proinflammatory cytokine IL-6, which negatively regulates the expression of cytochrome P450 [50] has not been changed.

The results obtained for the selective NMDA receptor GluN2B subunit antagonist CP-101,606 in our experiment differ from those of Loch et al. and Chan et al. [51,52] for the non-subunit-selective NMDA receptor antagonist ketamine. When administered in vivo to rats in a single dose of 10 mg/kg ip., ketamine only weakly decreased CYP2D and CYP3A activity [51], but when given at doses of 10–80 mg/kg ip. twice daily for 4 days, it induced CYP enzymes (CYP1A, 2B, 2E1 and 3A) after the highest dosage [52], which exceeded by far that applied in animal pharmacological tests to show antidepressant action [53]. The reason for this discrepancy may be related to different molecular mechanism of action within NMDA receptor (receptor subunit selectivity), the applied dosages and chemical structures of the two drugs, which may affect drug action on cytochrome P450 at the level of the neuroendocrine system and liver. It seems also worth mentioning that the distribution of particular mGluN2 subunits of NMDA receptor within the brain is different, which makes the action of CP-101,606 also brain region-dependent [35]. On the other hand, ketamine is metabolized to norketamine and hydroxynorketamine. The latter metabolite (*2R,6R*-HNK) acts at AMPA receptors and metabotropic mGlu_2_ receptors, broadening in this way the spectrum of ketamine action at glutamatergic receptors [54,55].

In conclusion, the obtained results imply the contribution of brain NMDA glutamate receptors to the neuroendocrine regulation of cytochrome P450 expression. However, further studies after intracerebral administration of CP-101,606 are necessary to confirm the central neuroendocrine regulation of cytochrome P450 by the applied selective antagonist of GluN2B subunit of NMDA receptor. It seems that there is a potential for metabolic drug–drug interactions with drugs acting on NMDA receptors.

## 4. Materials and Methods

### 4.1. Animals and Materials

All procedures involving animals and their care were conducted in conformity with the NIH Guide for the Care and Use of Laboratory Animals and the Ethics Committee of the Maj Institute of Pharmacology, Polish Academy of Sciences, Krakow. Adult male Wistar Han rats weighing 225–250 g were purchased from Charles River Laboratories (Sulzfeld, Germany). All animals were housed under controlled conditions with an artificial 12-h light/dark cycle (lights on from 07:00 to 19:00), 55 ± 5% humidity, and a temperature of 22 ± 2 °C. Food and tap water were freely available. To avoid any influence of the digestive process on the activity of CYP enzymes, eighteen hours before experiment, food was taken away from rats.

Axon Medchem (Groningen, The Netherlands) provided the selective NMDA receptor GluN2B subunit antagonist CP-101,606. Reagents for determining the activity of CYP enzymes (glucose-6-phosphate-dehydrogenase, glucose-6-phosphate, NADP and NADPH) were purchased from Sigma (St. Louis, MO, USA). Specific substrates and their metabolites were used to determine the activity of respective CYP enzymes: caffeine and 1,3,7-trimethyluric acid from Sigma (St. Louis, MO, USA), testosterone and its hydroxymetabolites from Steraloids (Newport, KY, USA), warfarin from Merck (Darmstadt, Germany), and 7-hydroxywarfarin synthesized at Maj Institute of Pharmacology, Polish Academy of Sciences [56]. RNA was isolated with mirVana kit from Life Technologies (Carlsbad, CA, USA). Transcriptor High-Fidelity cDNA synthesis kit was used (Roche Diagnostics, Indianapolis, IN, USA). TaqMan assays and the TaqMan Gene Expression Master Mix came from Life Technologies (Carlsbad, CA, USA). The ELISA kits for serum corticosterone from DRG MedTek (Warsaw, Poland), for serum T_3_ and T_4_ from Cloud-Clone Corp. (Katy, TX, USA), for pituitary growth hormone-releasing hormone (GHRH) from MyBiosource (San Diego, CA, USA), and for the interleukin IL-6 from R&D Systems (Minneapolis, USA) were used. The polyclonal primary anti-rat CYP1A, CYP3A1 and CYP3A2 antibodies from Millipore (Temecula, USA), anti-rat CYP2A antibody from Fine Test (Wuhan, Hubei, China), anti-rat CYP2C11 antibody from Abcam (Cambrige, UK), anti-rat CYP2C6 antibody from Gentest Corp. (Woburn, MA, USA), anti-rat CYP2B and anti-rat β-actin antibody from Santa Cruz (Dallas, TX, USA) were used. Rat cDNA-expressed CYP1A2, CYP2B1, CYP2C6, CYP2C11, CYP3A1 and CYP3A2 (Supersomes) were from Gentest Corp. (Woburn, MA, USA). The chemiluminescence reagents LumiGlo kit came from KPL (Gaithersburg, MD, USA).

### 4.2. Collection of Pituitary, Liver and Serum Samples

The selective NMDA receptor GluN2B subunit antagonist CP-101,606 was administered intraperitoneally in a pharmacologically active dose of 20 mg/kg [57] for 5 days or 3 weeks. The number of rats in the 5-day experiment was 10 for the control and 10 for the CP-101606-treated group. In the 3-week experiment, the control and CP-101,606-treated group consisted of 13 animals each. Two hours after the last injection of CP-101,606, the animals were decapitated, and pituitaries and livers were quickly isolated and stored at –80 °C. The blood serum was obtained by centrifugation and stored at −80 °C. Liver microsomes were prepared by differential centrifugation in standard conditions (homogenization in 20 mM Tris/KCl buffer of pH = 7.4, and next washing with 0.15 M KCl) as described previously [58].

### 4.3. Estimation of Cytochrome P450 Enzyme Activities in Rat Liver Microsomes

The activities of CYP enzymes in liver microsomes were studied using CYP-specific reactions conducted in the previously optimized conditions [56,59,60,61]. Briefly, the activity of CYP1A was determined as the rate of caffeine C-8-hydroxylation [59], the activity of CYP2C6 was assessed as the rate of warfarin 7-hydroxylation [56], while the activities of CYP2A, CYP2B, CYP2C11 and CYP3A were estimated as the rates of testosterone hydroxylation in positions 7-α, 16-β, 2-α and 16-α, and 2-β and 6-β, respectively [60,61,62]. The metabolites formed from specific substrates were quantified by HPLC with UV detection.

In the 5-day experiment, the results of CYP activity were obtained from 8–10 rats for the control and CP-101,606-treated group. In the 3-week study, the CYP activity was derived from 11–13 rats for the control and CP-101,606-treated group.

### 4.4. Estimation of the Cytochrome P450 Enzyme Protein Levels in Liver Microsomes

Western blot was performed as described previously [9]. Briefly, microsomal proteins (10 µg) were loaded on SDS gels in a Laemmli buffer system, and after separation transferred onto a nitrocellulose membrane. The blots were probed with primary antibodies (polyclonal rabbit anti-rat CYP1A, 2A, 2B, 2C6, 2C11, 3A1 or 3A2 antibodies), and then they were incubated with the appropriate horseradish peroxidase-conjugated secondary antibodies. The bands were visualized by enhanced chemiluminescence. Rat cDNA-expressed CYP enzymes (Supersomes): CYP1A2, 2A1, 2B1, 2C6, 2C11 (5 µg), 3A1 and 3A2 (1 µg) were used as standards. The immunoblots were evaluated using a luminescent image analyzer (LAS-1000) and Image Gauge 3.11 programs (Fuji Film, Tokyo, Japan). β-actin immunoreactivity was used to normalize the obtained data.

The results of the CYP proteins were obtained from 8 rats for the control and CP-101,606-treated group in the 5-day and 3-week experiment.

### 4.5. Determination of Hormone and Cytokine Levels

Hormonal responses were measured 2 h after the repeated administration of CP-101,606. The levels of hormones in serum or pituitary were measured using ELISA kits for GHRH, GH, corticosterone and thyroid hormones (T_3_, T_4_). The serum concentrations of cytokine were analyzed using the IL-6 ELISA kits. Absorbance was measured using a Synergy Mx Monochromator-Based Multi-Mode Microplate Reader (Biotek, Winooski, VT, USA).

The hormone and cytokine concentrations were calculated from 8–10 rats for the control and drug-treated group in the 5-day experiment or from 11–13 animals for the control and CP-101,606-treated group in the 3-week study.

### 4.6. RNA Isolation and Quantitative Real-Time Polymerase Chain Reaction (qRT-PCR)

The RT-qPCR method used in this study has been previously described in detail by Rysz et al. [9]. Briefly, RNA was extracted from individual liver samples using a mirVana isolation kit. RNA was reverse-transcribed using a Transcriptor High-Fidelity cDNA synthesis kit. Quantitative real-time PCR was performed in duplicate with TaqMan Gene Expression Assays using TaqMan Expression Master Mix, species-specific TaqMan type probes and primers (Table 2) and Bio-Rad CFX96 PCR system (Bio-Rad, Hercules, CA, USA). Real-time PCR was conducted under the standard conditions (50 °C for 2 min and 95 °C for 10 min followed by 40 cycles of 95 °C for 15 s and 60 °C for 1 min). The abundance of RNA was achieved by a comparative delta-delta Ct method (2^−∆∆Ct^). The expression of the two reference genes: β-actin *(ACTB)* and glyceraldehyde-3-phosphate dehydrogenase *(GAPDH*) was also measured. The obtained results concerning *CYP* mRNAs were normalized to the *ACTB* expression (*ACTB* expression was stable compared to that of *GAPDH* after CP-101,606 treatment).

The results of the *CYP* mRNA levels were calculated from 8–10 rats for the control and CP-101,606-treated group in the 5-day experiment or from 11–13 animals for the control and CP-101,606-treated group in the 3-week study.

### 4.7. Statistical Analysis

The obtained results are reported as the mean ± S.E.M. of 8–10 rats for the control and CP-101,606-treated group in the 5-day experiment or of 11–13 rats for the control and CP-101,606-treated group in the 3-week study, except for the western immunoblot analysis, where all the results were obtained from 8 animals for each group and experiment. Changes in the concentrations of hormones and interleukin, as well as the liver CYP enzyme activities, protein and mRNA levels, were statistically assessed using a two-tailed Student’s *t*-test. The changes found were considered as statistically significant when *p* < 0.05.

## 5. Conclusions

The presented results show a new possible physiological regulatory mechanism of liver cytochrome P450 involving the glutamatergic system. The obtained results imply the contribution of NMDA glutamate receptors to the neuroendocrine regulation of cytochrome P450 expression. They suggest that, apart from their targeted therapeutic effect as psychotropic drugs, new drugs acting via NMDA receptors may affect neuroendocrine regulation of physiological processes including the expression of cytochrome P450, in particular, during the first weeks of therapy. Thus different drug–drug interactions could occur in relation to the regulation of cytochrome P450 expression/activity via NMDA receptors during therapy with drugs acting on these receptors.

## Figures and Tables

**Table 1:** Summary of the effects of the 5-day and 3-week treatment with CP-101,606 on the expression and activity of liver cytochrome P450 enzymes.

CYPs	5 Days					3 Weeks				
	Activity	Protein		mRNA		Activity	Protein		mRNA	
1A1/2	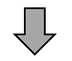	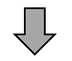		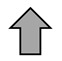	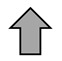	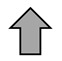	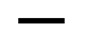		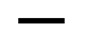	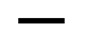
2A1/2	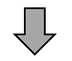	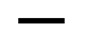		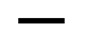	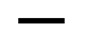	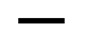	n.t.		n.t.	n.t.
2B1/2	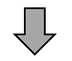	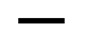		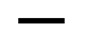	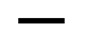	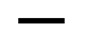	n.t.		n.t.	n.t.
2C6	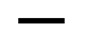	n.t.		n.t.		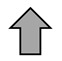	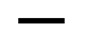		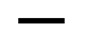	
2C11	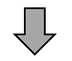	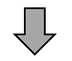		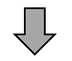		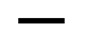	n.t.		n.t.	
3A1/2	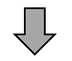	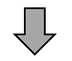		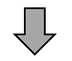		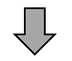	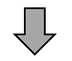	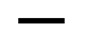	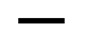	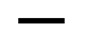

↑, ↓ increase or decrease, respectively; − no change; n.t. not tested.

**Table 2:** List of TaqMan^®^ Gene Expression Assays used in the study.

Gene Name	Assay ID
*CYP1A1*	Rn01418021_g1
*CYP1A2*	Rn00561082_m1
*CYP2A1*	Rn04219367_m1
*CYP2A2*	Rn00562207_m1
*CYP2B1*	Rn01457880_m1
*CYP2B2*	Rn02786833_m1
*CYP2C6*	Rn03417171_gH
*CYP2C11*	Rn01502203_m1
*CYP3A1*	Rn03062228_m1
*CYP3A2*	Rn00756461_m1
*ACTB*	Rn00667869_m1
*GAPDH*	Rn01462662_g1

## Data Availability

Data is contained within the article.
